# Improved spatial ecological sampling using open data and standardization: an example from malaria mosquito surveillance

**DOI:** 10.1098/rsif.2018.0941

**Published:** 2019-04-10

**Authors:** Luigi Sedda, Eric R. Lucas, Luc S. Djogbénou, Ako V. C. Edi, Alexander Egyir-Yawson, Bilali I. Kabula, Janet Midega, Eric Ochomo, David Weetman, Martin J. Donnelly

**Affiliations:** 1 Centre for Health Information, Computation and Statistics (CHICAS), Lancaster Medical School, Lancaster University, Furness Building, Lancaster LA1 4YG, UK; 2 Department of Vector Biology, Liverpool School of Tropical Medicine, Pembroke Place, Liverpool L3 5QA, UK; 3 Institut Régional de Santé Publique/Université d'Abomey–Calavi, BP 384 Ouidah, Benin; 4 Centre Suisse de Recherches Scientifiques en Cote d'Ivoire, 01 BP 1303 Abidjan 01, Cote d'Ivoire; 5 Department of Biomedical Sciences, University of Cape Coast, Cape Coast, Ghana; 6 National Institute for Medical Research (NIMR), Amani Centre, PO Box 81, Muheza, Tanzania; 7 Centre for Geographic Medicine Research, Kenya Medical Research Institute, PO Box 230, 80108 Kilifi, Kenya; 8 Centre for Global Health Research, Kenya Medical Research Institute, PO Box 1578 – 40100 Kisumu, Kenya; 9 Wellcome Sanger Institute, Hinxton, Cambridge CB10 1SA, UK

**Keywords:** remote sensing and field data, mosquito sampling, stratification, adaptive and non-adaptive sampling design, model-based geostatistics, Sub-Saharan Africa

## Abstract

Vector-borne disease control relies on efficient vector surveillance, mostly carried out using traps whose number and locations are often determined by expert opinion rather than a rigorous quantitative sampling design. In this work we propose a framework for ecological sampling design which in its preliminary stages can take into account environmental conditions obtained from open data (i.e. remote sensing and meteorological stations) not necessarily designed for ecological analysis. These environmental data are used to delimit the area into ecologically homogeneous strata. By employing Bayesian statistics within a model-based sampling design, the traps are deployed among the strata using a mixture of random and grid locations which allows balancing predictions and model-fitting accuracies. Sample sizes and the effect of ecological strata on sample sizes are estimated from previous mosquito sampling campaigns open data. Notably, we found that a configuration of 30 locations with four households each (120 samples) will have a similar accuracy in the predictions of mosquito abundance as 200 random samples. In addition, we show that random sampling independently from ecological strata, produces biased estimates of the mosquito abundance. Finally, we propose standardizing reporting of sampling designs to allow transparency and repetition/re-use in subsequent sampling campaigns.

## 1. Introduction

Sampling design is a crucial step in any survey as it affects the quality of data collection and analysis [1]. Sampling strategies should therefore be designed to maximize the effectiveness of the study, using any relevant preliminary and background data available [2]. Furthermore, because published sampling strategies frequently inspire designs for future studies, both the design details and justification should be rigorously reported. Despite improvement in recent years, both the use of available informative data and the rigour with which sampling designs are reported continue to fall short of what could be achieved [3]. Specifically, the amount of environmental data available from open-data platforms is often acknowledged but rarely exploited to support sampling design, while the necessary information for study repeatability, comparability or usability are often inadequately reported. Such data, even when not collected for ecological analyses, can support representativeness in investigations of population dynamics, epidemiological processes and biological studies. Here we use an example from malaria vector surveillance to design a sampling strategy for collecting mosquitoes for whole genome sequencing based monitoring and evaluation.

Genomic technologies are radically transforming our understanding of vector-borne disease transmission dynamics [4] due to the capacity to unveil complex interaction between human, pathogen, vector and environment. Whole genome sequencing projects have revealed novel genetic loci associated with increased susceptibility to malaria in the human host [5,6] and made major contributions to our understanding of how anti-malarial and insecticide resistance evolves [6]. However, the impact of environment on genotype distributions is much more poorly understood, reflecting at least in part the use of insufficiently ecologically informed sampling strategies. Much of the sampling conducted in vector surveillance studies is opportunistic and lacks a rigorous sampling framework. Often, ecological and entomological sampling designs rely solely on resource availability rather than aiming to maximize representativeness and precision of the variable of interest, e.g. collectors target locations where disease vectors are known to be abundant.

Designing a field sampling strategy requires three decisions: what is the variable of interest (formally the estimator, e.g. vector density), the sampling approach (e.g. model-based or not) and sampling location distribution (e.g. the number and spatial / temporal allocation of sampling points). These decisions constitute the sampling strategy trinity [7] in which each element strictly depends on the other two. Sampling strategies are further complicated by deterministic (e.g. due to age, environment, socio-economic, etc.) and stochastic (i.e. spatio-temporal autocorrelation) factors. Our literature search in Web of Science on spatial sampling of mosquitoes (search terms: mosquito OR anopheles AND sampling AND spatial, in title/keywords/abstract—last access in August 2018) shows that while all studies provide a general description of the sampling design, only a limited number of papers (i.e. [8–11]) give a detailed description of the rationale, decisions and calculations related to the ‘Where, When, How and How many’ samples to collect (see for example the reviews from [12] and [13]). In other literature, partial justification of the sampling design is provided. For example, [14–22] used previous surveillance information and remote sensing data to identify potential mosquito habitat types (or, in statistical terms, ‘strata’). However, the method used or assumptions made to choose the within-strata location and number of traps were not described, perhaps because these were entirely guided by practical considerations (i.e. [23]) or due to the high level of complexity or uncertainty in the scope of the sampling, which makes quantitatively-driven spatial sampling design very difficult (for example when the scope is describing a concurrent variety of species such as *Anopheles*, *Culex* and *Aedes*) [24,25]. Conversely, descriptions of sampling over time are often provided in detail, with explicit information on the frequency and length of the sampling campaign.

The picture that emerges from the literature is that using habitat stratification to inform sampling is a common procedure in vector biology, but often based on subjective or qualitative decisions. However, stratification has a fundamental role in describing and reducing the error in estimates of mosquito variation, which in turn influences surveillance success, assessment of epidemiological risk and genetic diversity [26]*.* Identifying a set of (independent) environmental variables homogeneous within strata allows a better representation and representativeness of the environment related to the property or properties under study (i.e. insect abundance and insecticide resistance) [13]. Unless the spatial or spatio-temporal autocorrelation of the property under study is tested and found negligible [27], these approaches often incorrectly assume independence between samples in space and time [28], which is an unrealistic assumption for most of the ecological processes. Spatial and spatio-temporal heterogeneity can be accounted for in sampling design by adopting a geostatistical model-based sampling design [8,29].

Ecological stratification of sampling designs is now facilitated by web-based open data providers, allowing rapid access to large amounts of information on climate and land-use, which are commonly associated with biogeographic patterns of human and animal health and species distribution [30]. This availability of open data (largely remote sensing) for almost every global location, combined with appropriate spatio-temporal algorithms [15], make quantitative ecological stratification more accessible as a preliminary step to any sampling programme. Nevertheless ‘*very few studies propose, at an early phase of research work, objective sampling strategies that are consistent with both study goals and constraints*’ [13]. In this work we propose a framework for optimizing the sampling design of the spatial distribution of mosquito populations using open data, which we hope will be relevant to a wide range of ecological, disease monitoring and genomic studies.

This paper describes the stages constituting our sampling framework which is based on the following decisions:

1. The variable of interest. In our case is the presence of insecticide resistance genes in the mosquito genomes. To achieve this we will trap mosquitoes in areas with known or suspected insecticide resistance.
2. The sampling approach. The sample size is calculated based on previous mosquito surveys, and sample locations are defined to balance prediction and parametrization, i.e. the accuracy in predictions and the goodness of model fitting when limited amount of information of the variable of interest is available.
3. The stratification. The open data are used to ecologically characterize the area(s) under study and inform the location of each trap. The effect of ecological strata on sampling size is estimated from a previous malaria control surveillance campaign.

Finally, we discuss the necessity and benefits of a standardization of the sampling design procedures and reports to make them repeatable and reusable.

## 2. Materials

### 2.1. GAARDian project

The sampling design described in this work has been developed within the UK-MRC-funded GAARDian project (https://www.anophelesgenomics.org/gaardian). The main objective of this project is to investigate the spatial and temporal scale of variation in mosquito genomes to improve our understanding of the processes underlying the spread of insecticide resistance. Insecticide resistance is a major threat to the sustained control of malaria, as 260 million averted clinical cases of malaria have been due to the use of insecticides that target the mosquito vector [31].

### 2.2. Study area

**Figure 1.**
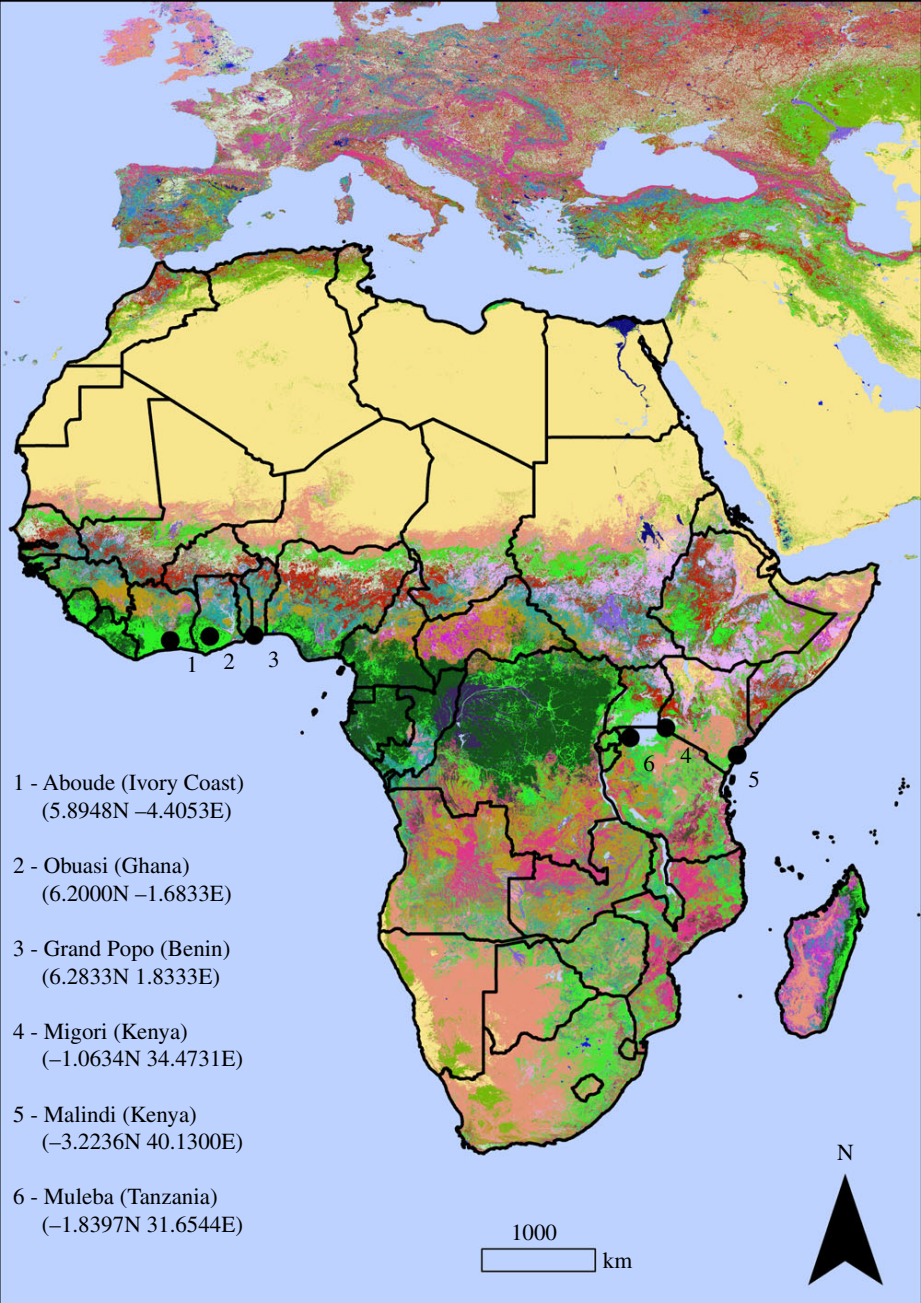
Location of the GAARDian sampling sites, shown on a land cover background (GlobeLand30 land covers). Map was made using ArcMap 10.4 (http://desktop.arcgis.com/en/arcmap/). Source administrative limits: http://www.maplibrary.org/library/index.htm. (Online version in colour.)

Six sites were chosen based on suspected use of insecticide or presence of insecticide resistance (figure 1) (see electronic supplementary material, appendix A, for detailed description of each site). Around each site, an operable area was determined as the largest area where traps can be deployed and routinely checked by two operators. The operable area was a 60 × 60 km square centred on the site.

### 2.3. Environmental data

We used open data information from several sources to stratify the ecological variations of each study site. These data include land cover, climate and topography [32].

GlobeLand30 (http://www.globallandcover.com/GLC30Download/index.aspx) is a global land cover map of 30 m resolution produced by the National Geomatics Center of China and containing 10 land cover classes (full description of classes in [33]). The images used for GlobeLand30 classification are multispectral images, including the TM5 and ETM+ of America Land Resources Satellite (Landsat) and the multispectral images of China Environmental Disaster Alleviation Satellite (HJ-1). GlobeLand30 raster adopts WGS84 coordinate system, UTM projection, 6-degree zoning and the reference ellipsoid is WGS 84 ellipsoid.

The moderate-resolution imaging spectroradiometer (MODIS) satellite products are provided in monthly time-series at 0.05 degree (approx. 5 km) resolution from observations by the MODIS sensor on Terra (AM) for the period February 2000 to December 2013 inclusive and available at (https://ora.ox.ac.uk/objects/uuid:896bf37f-a56b-4bc0-9595-8c9201161973) [34]. The following MODIS products were used:

— MODIS Enhanced Vegetation Index (EVI) from the MOD13C2 product comprises monthly, global EVI. This resource provides consistent spatial and temporal comparisons of vegetation canopy greenness, a composite property of leaf area, quantity of chlorophyll and canopy structure. EVI improves sensitivity over dense vegetation conditions or heterogeneous landscapes when compared to Normalized Difference Vegetation Index (NDVI).
— MODIS Air Temperature (Temp) from the MOD07_L2 Atmospheric Profile product comprises monthly, global temperature at the closest level to the earth's surface.
— MODIS Evapotranspiration (ET) from the MOD16 Global Evapotranspiration product is calculated monthly as the ratio of Actual to Potential Evapotranspiration (AET/PET).

Precipitation was obtained from WorldClim v. 2 as average annual precipitation from 1970 to 2000 at 30 arcseconds (1 km^2^ *ca*) (http://worldclim.org/version2) [35]. Finally, elevation was obtained from the NASA Shuttle Radar Topographic Mission (SRTM) 90 m Digital Elevation Database v. 4.1. The SRTM 90 m DEM's have a resolution of 90 m at the equator. The DEM is available in geographical coordinate system—WGS84 datum (https://drive.google.com/drive/folders/0B_J08t5spvd8VWJPbTB3anNHamc).

## 3. Methods

Defining a standard spatial sampling design does not affect the multitude of choices necessary for each different problem, but it requires that three elements are fully described: sample size; stratification (if stratification is performed) and geographical allocation of the sampling points. A description of each element optimization is given below.

### 3.1. Sampling size optimization

For one of the collection areas (Migori, Kenya, location 4 in figure 1), additional data were available from entomological surveillance carried out from December 2015 to September 2017 as part of indoor residual spraying (IRS) (Abong'o *et al.* 2018, unpublished; http://www.africairs.net/about-airs/), which we will refer to as AIRS data hereafter. As in the GAARDian project, collections were made using CDC light traps [36], hung in each house over the sleeping area, approximately 1.5 m from the ground, adjacent to an occupied bed net. The traps were run from 18.00 and mosquitoes were collected at 07.00 the next morning. Placing the trap near sleeping space facilitates sampling female mosquitoes that are actively seeking a blood meal. We used this preliminary information about mosquito abundance to estimate the optimal sample size (in terms of mosquito distribution) to be used in all sites.

From the AIRS data, we first estimated the spatial covariance function (via maximum-likelihood estimation, [37]) that was used to simulate a log Gaussian Cox process (LGCP) [38] mimicking the mosquito spatial distribution process found in Migori. This can be translated in lay words as a process (mosquito catches) that is environmentally driven but producing values of catches that can be considered independent (i.e. catch on one occasion does not predict subsequent catches in the same or nearby locations) although the average process is spatially dependent (hence the necessity to estimate the spatial covariance function above).

The Gaussian random field is of the form [39]

3.1 y(l)=μ(l)+Z(l)+ε,

where *l* is the location, *μ* is the mean, **Z** is the Gaussian process with Matern correlation function, and *ɛ* is the error term (noise or nugget).

The Matern correlation function has the general form

3.2 $$Z(l)=\frac{1}{2^{h-1}\Gamma \;(h)}{\left(\frac{2l\sqrt{h}}{r}\right)}^{h}K_{h}\left(\frac{2l\sqrt{h}}{r}\right),$$

where **K***_h_*(·) is the modified Bessel function of order *h* and *r* is the spatial range [40]. Both *h* and *r* must be positive and different from 0.

Finally the Poisson LGCP can be written as [41]

3.3 Y(l)∼P(λ(l))

and

3.4 λ(l)=exp(y(l)),

where *Y* is the mosquito density point process and *λ* is the conditional mean. As can be easily noted, equation (3.4) links directly to equation (3.1).

From the LGCP we predicted the estimated variance in the parameters of the spatial covariance function and the prediction error for a set of sample sizes (15, 30, 75, 150, 200 and 300) assumed randomly allocated in the area of Migori.

This will allow the allocation of the (limited) resources to obtain the sample size that will produce the desired prediction error and variance in the spatial covariance parameters (if this is an objective of the sampling design).

### 3.2. Stratification (ecological delineation)

In many areas of physical, engineering, life and social sciences, inferential and predictive classification are prevalent tools to discriminate between classes and to interpret the differences. Examples range from identification of ecological niches to brain and bone anomalies. While the growing amount of open access information enables discrimination among a large number of ecological classes, many traditional algorithms fail for these data because of decreased classification performance (leading to overfitting) and mathematical/practical limitations [42]. One method is to describe ecological strata in terms of transformed environmental variation (i.e. factorial analyses) [13], but the results can be difficult to interpret. By contrast, discriminant analysis (DA) requires less computational time and resources because no parameter tuning is required [43]. Discriminant analysis [44] is a common multivariate statistical approach for data classification (for example, in 2017 2026 scientific articles were published on the use or improvement of discriminant analysis—search terms used in Web of Science: ‘discriminant analysis’, in title/keywords/abstract).

The simplest forms of DA are linear (LDA) and quadratic (QDA). LDA can be seen as a regression line whose orientation divides a high-dimensional space, reducing the dimensionality while keeping each class separate from the other classes. In practice, the optimal orientation is the one that minimizes the within-class variance and maximizes the between-class variance [45]. The main assumption of LDA is that all the classes have a common variance–covariance matrix, i.e. the relationships between classes and explanatory variables are independent from class membership, while the differences between classes are dependent only on the mean.

When the variance–covariance matrices are not homogeneous for two or more classes, linear discriminant analysis cannot be applied. Instead the QDA can be employed. The QDA discriminant function is

3.5 $$f_{i}^{Q}=-\frac{1}{2}\log|\Sigma_{i}|-\frac{1}{2}({\left(X-\mu_{i}\right)}^{T}\Sigma_{i}^{-1}(X-\mu_{i}))+\log(\;p_{i}),$$

where **X** is the matrix of variables, *μ* the vector containing the mean of each variable and **Σ** is the variance–covariance matrix, and *p_i_* the ‘prior’ probability of each point to belong to the class *i*. *i* is the subscript for class *i*, with *i* = 1, …, *N* where *N* is the total number of classes.

*f* is calculated based on a training dataset (class memberships are known). The larger the *f* value, the higher the probability that the point belongs to that group. For a training dataset, the *p_i_* can be calculated in several ways, usually by ‘equal priors’ method: each class has a prior probability equal to 1/*N*. In this analysis, and in order to take into account the spatial proximity of the classes, a local frequency prior method was used. It estimates the class *p_i_* prior probability as the relative frequency of *i* labels in the neighbourhood. Similarly, predicting a label for a new point means looking at the local proportion of each class (as classified from the training dataset) around the new point.

Once *f* is maximized with the training dataset, a new data point can be classified by calculating *f* for the new point and for each class (equivalent to calculating the position of a point with respect to all available class centroids), and assigning to it the class index at which corresponds the maximum *f*.

The QDA has been embedded into an algorithm that determines the optimal number of ecological classes and their geographical delimitation for each area (see electronic supplementary material, appendix B, for further details).

The analysis was carried out taking all the environmental variables at their original spatial resolution, and providing the output (classification) at 30 m resolution (the same as the land cover resolution).

### 3.3. Spatial allocation of the sample households

For the present study the malaria vector species we are targeting, within the *Anopheles gambiae* species complex, are usually highly anthropophilic and commonly found in houses. Therefore, the traps are located inside households.

Locations of the sampling points, in each sampling site (figure 1), follow a ‘lattice plus close-pairs’ design [46] which combines regular lattice (efficient for predictions) and random points as close pairs (efficient for parameter estimation) [47].

For an easier understanding of the sampling design, we refer to the six locations distributed in West and East Africa as sampling sites (shown in figure 1). Each sampling site will contain *M* sampling points. Each sampling point contains *V* households. Therefore, the total number of households sampled per sampling site is *M* × *V*.

**Figure 2.**
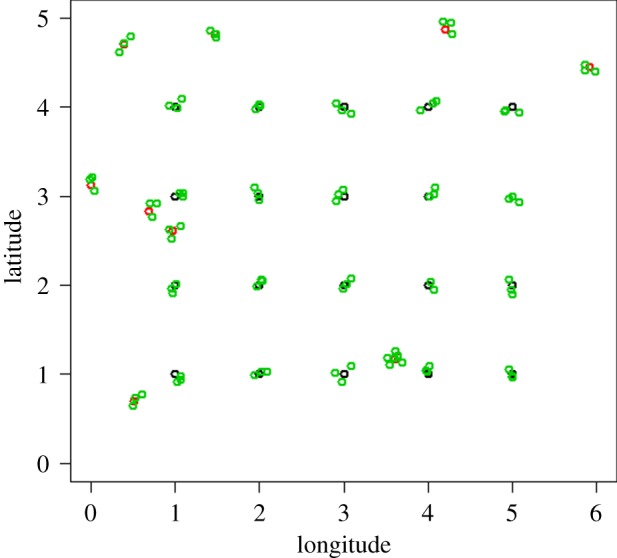
Example of lattice with close pairs design adopted in this work. Black dots, sampling locations in regular grid (the 4 rows x 5 column grid at the centre of the figure); red dots, sampling locations allocated randomly (noticeable because they don't follow the grid); and green dots are the households identified sufficiently close to the sampling locations (*V*) (identified with the three clustered dots at each grid and random sampling location). Plot was made using R-cran 3.5.0 (http://r-project.org). (Online version in colour.)

For all sites except Migori, the distribution of *M* sampling points are realized under two conditions: (i) 70% of sampling points are in grid (lattice) and 30% close-pairs randomly allocated (a proportion usually applied in simulation analyses, i.e. [46] and [48]) (figure 2); (ii) each stratum must contain a number of points proportional to the stratum size [49]:

3.6 $$n_{i}=M\frac{A_{i}}{A_{T}},$$

where *n_i_* is the number of points for class *i*; *A_i_* the area of class *i;* and *A*_T_ is the total area. The term ‘close pairs’ here is used loosely, since not all the points in the grid will have a close pair, and some close pairs may be shared between points.

In Migori alone (where a previous sampling campaign, AIRS, took place) an adaptive sampling design was trialled in which AIRS sampling served to inform the location of the *M* sampling points. From the LGCP model (see above), we estimated the prediction variances at each grid cell, and attributed the *M* locations to the cells with highest prediction variance (uncertainty) [29,50–52].

### 3.4. Effect of stratification on sample size and improvement of mosquito abundance models

In order to evaluate the effect of stratification:

(a) on the ratio between the Poisson rate parameter of mosquito counts from a survey (*λ*_1_) and the Poisson rate parameter of mosquito count from a sub-sample of it (*λ*_2_) (random versus stratified);
(b) and on the goodness of fitting of mosquito abundance models;

we have considered a mosquito sampling campaign from Uganda. This data is from 104 health subdistricts (HSD) where estimates of *A. gambiae* and *Anopheles funestus* densities (as determined by a standard collection method) for both male and female mosquitos are available. The sampling design was based on a cluster randomized trial with 10 houses selected at random from each HSD, and mosquito collection was made every six months for 2 years (http://www.isrctn.com/ISRCTN17516395).

For objective (a) we have employed a Poisson exact text on the null hypothesis that the ratio between *λ*_1_ (obtained from the entire Uganda mosquito collection data) and *λ*_2_ (obtained from a sub-sample of mosquito collections of the Uganda data) is equal to 1, i.e. the two conditional means are not different [53]. The test is performed by first randomly sampling 2, 3, 4 and 5 mosquito collection locations from each strata. For each of these sample sizes the *λ*_2_ is calculated and the test performed. The process has been repeated 999 times, to randomize the location selection, and 95% confidence interval from the all bootstrapping are estimated. The procedure above was then compared with a sampling design that randomly extracts the same amount of locations from the entire dataset but independently from the strata to which they belong.

For objective (b) we fitted the total number of mosquitoes for each species and at each location (over the two years of collection) using the ecological strata produced by performing the same methodology described in the above section ‘Stratification’. The model fitting employs a Poisson generalized linear model [53] and model comparison against the null model is performed using a MANOVA test [18].

## 4. Results

### 4.1. Sample size

In order to estimate the impact of sample size on model fitting and predictions, we simulated a log Gaussian Cox Process with known covariance function. The latter has been parametrized with the AIRS mosquito surveillance data via maximum-likelihood estimation. The obtained LGCP parameters were: intercept of 21.77, spatial variance (sill—i.e. the amount of variance dependent on distance) of 14 478, spatial range of 16 km (i.e. the maximum distance at which variance increases with distance) and 0 nugget (variance independent of distance that can be due to measurement errors or un-explained factors). Therefore, according to the model, all the variation is considered to be spatially-dependent up to a 16 km range. Finally, the Matern kappa parameter (shape parameter) was 1.5 (see electronic supplementary material, appendix C).

The simulation results of the above model with different sample size are reported in table 1. With 30 sampling locations, the prediction error and the total variance in the LGCP parameters was halved compared to 15 locations, and 20 times less when using 75 locations. The standard error in predictions is the maximum number of mosquitoes predicted in excess or in deficiency to the true mean. Therefore with 30 traps it is estimated a maximum error of 93 mosquitoes around the real mean and with 200 locations an error of 22 mosquitoes.

**Table 1.** Total variance in the parameters of the Gaussian process (intercept, sill, nugget, range) and standard errors for the predictions at different sample sizes.

sample size	total variance in the parameter of the Gaussian process	standard error in predictions
15	8034	194
30	4726	93
75	454	44
150	71	31
200	14	22
300	0.86	9

In order to improve local estimates, more than one household within 2.5 km from each sampling point can be employed. Therefore if we take two households for each of the 30 sampling points, the total number of households is 60. The effect of the use of more than one household in model fitting and prediction is shown in table 2.

**Table 2.** Total variance in the parameters of the Gaussian process (intercept, sill, nugget, range) and standard errors for the predictions at different number of households at each sampling point, with 30 sampling points.

number of households at each sampling point	total variance in the parameter of the Gaussian process	standard error in predictions
2	5207	71
3	2073	61
4	1851	22
5	701	19
6	605	19
7	118	18

With four households and 30 sampling points we expect the same prediction error as using 200 random sampling points distributed across the entire area and each containing a single household (comparison of standard errors in tables 1 and 2) but higher variance in the parameters. Using between five and seven households has little impact on the standard error in the predictions, although there is a significant improvement in the model fitting as the number of households increases (see total variance column in table 2). Consequently, the sampling design was chosen with 30 locations and four households which was considered a good balance in terms of standard errors, model fitting and economic feasibility.

### 4.2. Ecological classification

**Figure 3.**
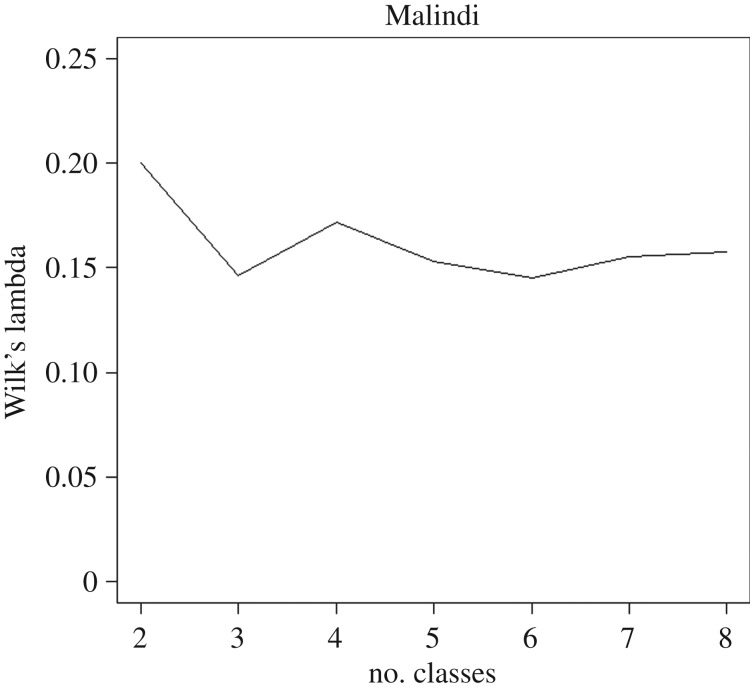
Wilk's Lambda criterion for Malindi. Graph was made using R-cran 3.5.0 (http://r-project.org).

The stratification identified two ecological classes for Migori, Obuasi, Muleba and Aboude; three ecological classes in Malindi and four in Grand Popo. The Wilk's criterion, measured as Wilk's Lambda, for Malindi is shown in figure 3; for other sites, they are provided in the electronic supplementary material (appendix D). The biggest improvement (i.e. largest decrease of the Wilk's Lambda) is in the change from 2 to 3 classes (figure 3).

**Figure 4.**
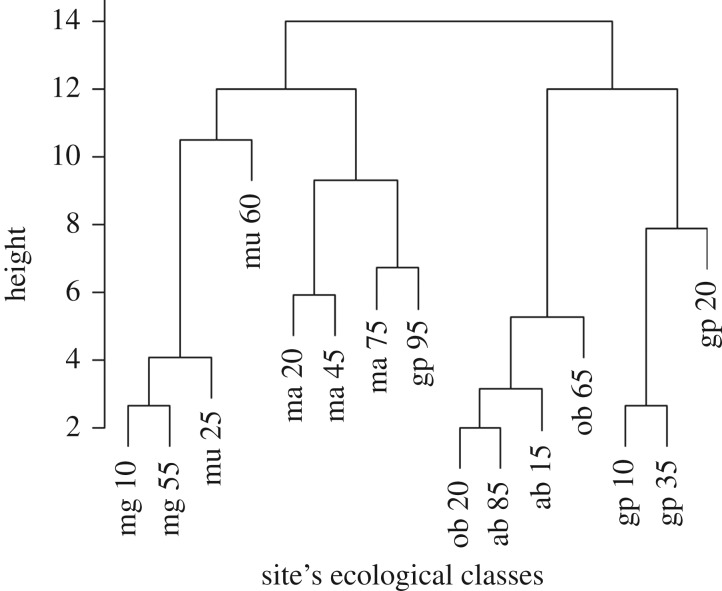
Dendrogram of agglomerative hierarchical clustering of the ecological zones. mg, Migori; mu, Muleba; ma, Malindi; gp, Grand Popo; ob, Obuasi; and ab, Aboude. For the description of the class number see electronic supplementary material, appendix E. Graph was made using R-cran 3.5.0 (http://r-project.org).

A hierarchical numerical classification of the sites and classes is shown in figure 4. This dendrogram was obtained from the agglomerative method (classes are aggregated into progressively larger groups) group average [54]. The latter accounts for the average distances or similarities between all the members of the new group and those of the others. The heights in figure 4 represent the dissimilarities between classes (classes full description reported in electronic supplementary material, appendix E), which are very small for some intra-location comparisons (mg10 and mg55 in Migori (Kenya); and gp10 and gp35 in Grand Popo (Benin)) and indeed some inter-country comparisons (ob20 in Obuasi (Ghana) with ab85 in Aboude (Cote d'Ivoire)). The classification suggests geographical homogeneity for most of the sites, since close locations looks more similar (Migori and Muleba, Obuasi and Aboude) than same classes far apart. For example, Malindi and Grand Popo classes (with exception of class 95 in Grand Popo) form their own clusters. Both Grand Popo and Malindi are coastal sampling locations, albeit on opposite sides of the African continent.

**Figure 5.**
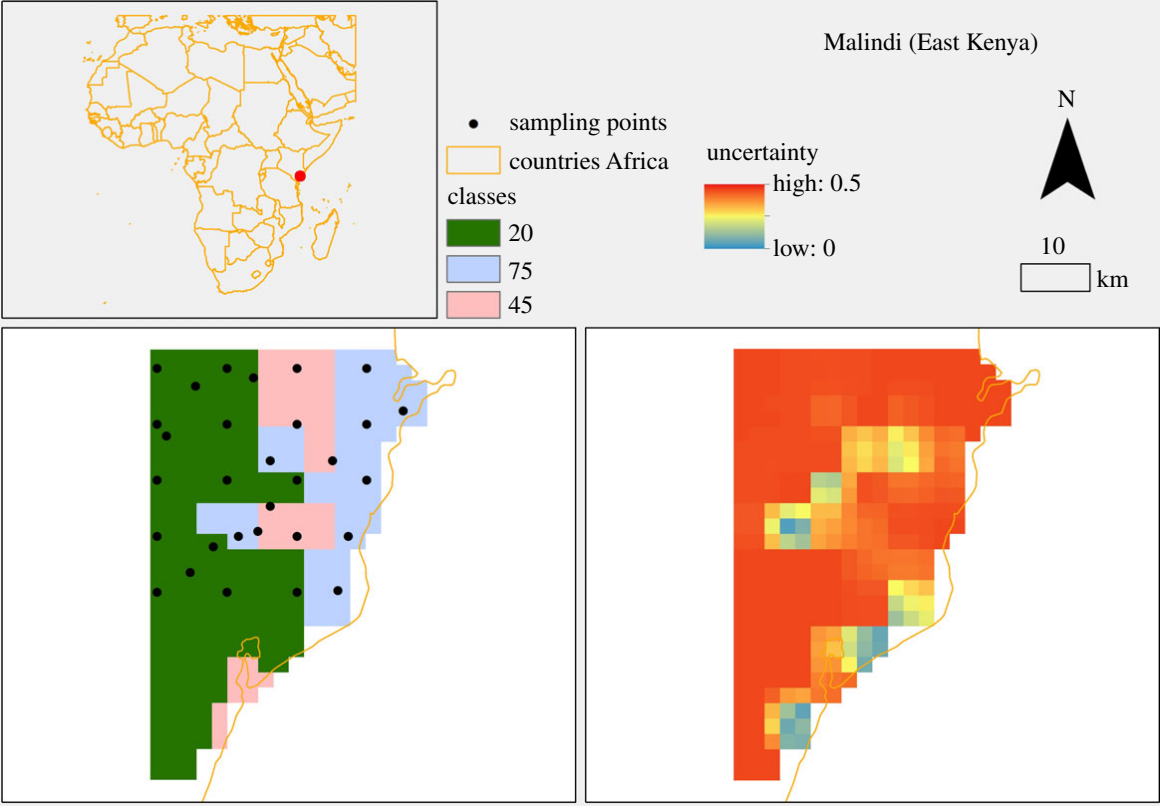
Ecological classification and uncertainty for the area of Malindi. Map was made using ArcMap 10.4 (http://desktop.arcgis.com/en/arcmap/). Source administrative limits: http://www.maplibrary.org/library/index.htm. (Online version in colour.)

Figure 5 shows the ecological classification and its uncertainty for the Malindi area. The same maps for the rest of the sites are shown in the electronic supplementary material (appendix F).

### 4.3. Sample locations

In figure 5 the sampling locations for Malindi are shown overlaid on the ecological classes. These locations are obtained from the lattice with close pairs sampling design, with a batch of four households (not shown in figure 5) at each sampling point. In this lattice with close pairs sampling design, 20 of the 30 locations are deployed in a 4 × 5 regular grid, and the rest allocated randomly as described in the methods. This general objective was modified to weight the number of locations by ecological classes; thus some of the points in the grid may have been adjusted slightly to be contained in the new class. In all the sites, each of these 30 locations constitutes a cluster of four households as in figure 2.

**Figure 6.**
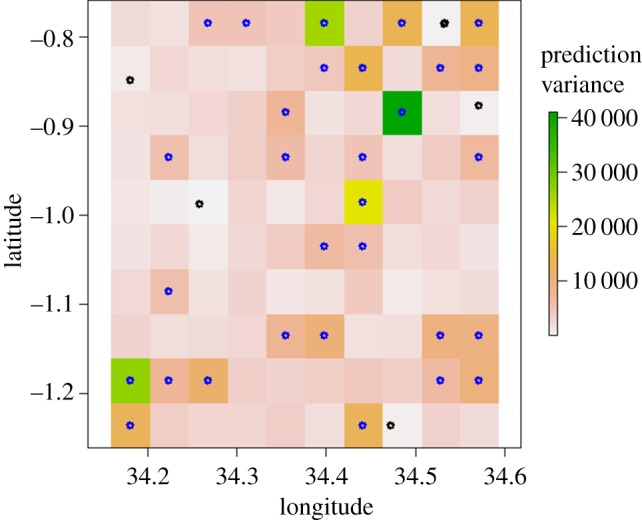
Adaptive sampling for Migori in class 10. Black dots are the AIRS mosquito surveillance locations. The grey dots (blue on the online version) are the adaptive locations, which are targeting the cells with largest prediction variance. Graph was made using R-cran 3.5.0 (http://r-project.org). (Online version in colour.)

The sampling location in Migori followed an adaptive sampling design, in which only class 10 was sampled (see electronic supplementary material, appendix F) because of the constraint that previous samples (AIRS) only targeted this class. The allocation of 30 samples and households in class 10 in Migori were based on the prediction variance, i.e. new sampling points are allocated at the centre of 30 pixels with largest prediction variance [52] (figure 6).

### 4.4. Effect of stratification on sample size and improvement of mosquito abundance models

For this objective we have used the Uganda dataset (see methods), which contains a large mosquito sampling campaign (104 districts, 1040 households) carried out over for 2 years. The first step was to identify the ecological strata for each cluster (of households) location. By applying the same stratification method described for here (see methods) we identified four ecological zones.

The stratification was first used to evaluate if a sub-sample (up to 20% of the full dataset) of the mosquito collections, extracted from each strata, is still representative of the full mosquito collection obtained in Uganda. Stratification produced ratios that are not significantly different from 1 (i.e. *λ*_1_ and *λ*_2_ are not significantly different) for any sample size (see electronic supplementary material, appendix G, table G1). In contrast for random sampling all ratios between the two rate parameters are significantly higher than 1 for all the sample sizes (i.e. *λ*_1_ and *λ*_2_ are significantly different). Finally stratification improves the model fitting of mosquito counts when compared to a null model (see electronic supplementary material, appendix G, table G2).

## 5. Discussion

Vector-borne disease control and monitoring rely on vector surveillance, mostly carried out using trap-based indices and, more recently, remote sensing data [13,26]. Trap-based indices (density, population changes, distribution, etc.) are calculated from mosquito catches and require a system of traps dispersed in the field in sufficient numbers to represent mosquito population ecology and dynamics. Conversely, remote sensing data can be used to define the ecological level of disease risk based on mosquito ecological suitability [49]. This is a cheaper and quicker option, but may not have the spatial and temporal resolution necessary for practical interventions [55]. In a sampling design, trap-based indices and remote sensing analyses must be seen as complementary tools, since remote sensing data contain the information necessary to define location and distance between sampling points.

Sample size, location, estimator and strategy (i.e. model based or not, adaptive or not) are the fundamental characteristics of a study design [27], which affect the likely success of describing the studied process (e.g. disease or organism distribution and abundance), its stochasticity, and, consequently, the accuracy of the estimates.

Ecologists are now equipped with algorithms, open information and datasets that enable a better understanding of the biology and spatial distribution of populations, which allows optimization of collection site placement to best describe natural processes. Ecological/environmental classification is now possible for every region in the world [55]. Failure to exploit these data in ecological and genomic sampling frameworks ignores the spatial variability of favourable, unfavourable or neutral habitats, therefore random or transect sampling designs may or may not be representative of the ground conditions and characteristics [56]. Even a grid design can be biased towards larger ecological classes and may miss linear features (i.e. a river passing between collection points) [1]. The consequence of which could be an over- or under-estimate of the true abundance, even when the population phenology is correctly delineated [56]. In some cases, however, a quantitatively determined sampling design may not be necessary, especially if the study intends to survey the entire area under study (as for example in [57,58]), or if it is a consequence of interventions recommended by national or international authorities [58,59] (i.e. WHO guidelines [60]).

In the illustrative example presented we demonstrate how these approaches may be used to develop an *a priori* sampling strategy to sample malaria vectors for genomic and ecological studies. The ecological classification presented for each site returned a maximum uncertainty ranging from 0.37 to 0.44 depending on the site (figure 5 for Malindi and electronic supplementary material, appendix F, for the other sites), which can be interpreted as the probability that a grid node belongs to a different class. This level of uncertainty shows that classification identified dominant classes. In addition, the ecological classification also shows that areas (the six sampling sites) with putatively the same land cover are still ecologically different when considering the full set of environmental variables (temperature, precipitation, elevation, evapo-transpiration and vegetation), and that geographical proximity is a dominant factor in ecological clustering [61] (figure 4). It is therefore not surprising that sites cluster much more strongly within country than within ecotype; e.g. forest (class 20) in Malindi (Kenya) is not equivalent to forest in Obuasi (Ghana) or Grand Popo (Benin). The ecological classes, while not often used in modelling mosquito populations and communities for medium- and large-scale analyses, represent the complex interaction of environmental and socio-economic conditions [23].

Another factor that we accounted for during our sampling design is the spatial autocorrelation of mosquito catches (model-based sampling design) [12]. The effect of strong autocorrelation can reduce the overall statistical power (and the overall biological significance of the study) as it results in effectively a lower sample size (because the assumption of independence is violated), underestimates of variance, and increases in type I error [10]. Geostatistical approaches, such as the one applied here, can lead to unbiased estimates of population parameters and avoid the risks and limitations of random, or haphazard, selection of sampling locations. Given the requirements to satisfy both parametrization and predictions [47], the simulated inhibitory design adapted from [50] in order to contain clusters of households at each sampling point, has shown that with 120 sampling houses for each site distributed across 30 sampling points, we achieve the same prediction error (main goal) as from 200 points allocated at random, albeit at the expense of parameter accuracy. However, there were important limitations in the sample size/location calculation. Firstly, they are based on limited pre-existing mosquito surveillance data from Migori, which may not describe the different spatial scales of the mosquito abundance distribution [26]. This is a concern due to the large variation in abundance levels observed throughout the period, but that can be solved by deploying an adaptive sampling design, i.e. concentrating the new samples where we have the largest uncertainties (our knowledge is poor) in the process of interest (abundance or a level of abundance). In addition, we are assuming that the mosquito population dynamics in Migori are similar to those in the other sites. The ecological classification has the advantage of correcting for local mosquito population dynamics although this is not a full solution. Ideally, Migori could have been used to analyse the effect of the ecological classification on mosquito estimates. Unfortunately, the pre-existing surveillance samples are located in the same ecological zone (electronic supplementary material, appendix F) making it impossible to simulate the effect of the ecological classification on the sample size/location optimization. For this reason we evaluated the effect of stratification on sample size using a different dataset (Uganda). This shows that 10–20% of mosquito collections randomly selected from strata are representative of the full survey. This result shows that stratification can be applied at any stage of the sampling campaign, and even if it was not considered at the initial (planning) phase, it can adaptatively inform the subsequent sampling phases or collections and optimize the sampling costs (subsequent sub-sampling of each strata). In addition, using the Uganda dataset, we have also shown that the ecological stratification improves model fitting, again representing a model feature that can be applied in both pre-analysis and post-analysis of sampling campaigns.

An element not considered in this analysis but that requires discussion is the temporal frequency and length of the sampling campaign. Designs for temporal sampling raise the same challenges as spatial designs, along with additional considerations. These include:

*is it better to trap six times in each of two houses, or twice in each of six houses, or four times in each of three houses? And in the latter case, is it necessary that the nights should be at weekly intervals, or would the easier task of sampling over four consecutive nights yield a similar amount of information? Should the same ‘fixed’ houses be sampled on each occasion, or should a new set be chosen randomly on each occasion?* (extracted from [62])

Answering these questions requires relatively lengthy longitudinal studies and a knowledge of *Anopheles* population dynamics. Fortnightly collections are common in mosquito sampling designs, and enable cost-effective descriptions of seasonality and variation in mosquito abundance [18]. On the other hand, positioning traps during peaks of mosquito abundance can significantly overestimate the rate of population increase and the level of abundance, and only sampling over two or more years may accurately account for cyclical fluctuations in vector abundance [62].

Our analysis provides an example of how to fully describe the assumptions, conditions and constraints of sampling strategies. We do not expect other researchers to precisely replicate our methodology, e.g. the use of four houses in 30 sampling locations depends on previous abundance analysis and may change when more information will be available (adaptive sampling). Instead we have shown how open-data sources and ecological information can be implemented in the initial steps of sampling design. Our literature review shows that the specifics of sampling design are poorly reported and we therefore suggest that even when sampling is based on expert-opinion decisions, a full description of the sampling design should be provided to make the sampling repeatable or comparable or usable for subsequent similar studies. For example, field constraints such as presence of the disease or vector or host, vegetation type and density, elevation, field hostility, logistic feasibility, potential interference, human proximity, breeding sites, and risk of trapping material theft [13] or the type of trap used [63], which often are the major influences in the sampling design, need to be declared and described. In fact, previous sampling campaigns are often used to inform future sampling design, and therefore standardization of sampling designs and protocols are now a priority.

Not only malaria studies lack sampling design information. For mosquito-borne arboviruses such as Dengue and West Nile Virus, or indeed other vectors such as ticks [64], sample size and locations can be solely based on economic and environmental constraints and/or expert-based decisions (see for example [65,66]), including national and sub-national sampling campaigns. In the Netherlands a national mosquito surveillance campaign [67] did stratify the sampling area based on land use and public health concerns (i.e. preferential sampling by oversampling urban areas) with number of locations depending on the pre-determined scale of the analysis. A similar, although less detailed, approach was taken in North Italy [68] for West Nile Virus. However, a recent study [69], employed an *a priori* G*Power analysis to determine the sample size, and then allocated the samples to (a) maximize the spatial spreading of mosquito sample sites, and (b) to sample at locations that would reflect a large range of socioeconomic conditions, since the objective was to estimate the effect of socio-economic drivers in *Aedes albopictus* distribution. While conceptually similar to the one proposed here (although our goal was to find ecological homogeneous areas instead of mapping socioeconomic differences), the authors do not consider the spatial autocorrelation in their sample size and design (which for diseases transmission by *Aedes* species is very important, see for example [27] and [70]), although they do use a maximum coverage approach.

Finally, our framework can be applied to other ecological studies. For example, Wang and colleagues [71] included a spatial autocorrelation index to improve the sampling design for crop acreage. This approach, however, does not model the spatial autocorrelation that is necessary to allocate spatial samples but simply tries to achieve independence between samples. Our framework may support systematic sampling when affected by spatial autocorrelation [72] or when geostatistical mapping is required [73].

In conclusion, big and open data and research outputs could enhance the power of ecological and genomic studies [3], facilitating the growth of complex and multidimensional algorithms. In the specific field of vector biology and genomics, there is an urgent need to establish standards for mosquito sampling design and description in scientific reports. One of the first steps is to facilitate training and workshops [11] but also the improvement of publishing standards (i.e. requiring authors to fully disclose the sampling design) in order to produce a collection of high quality and usable sampling designs along with their results.

## Supplementary Material

Electronic Supplementary Material

## Data accessibility

All data used in this research are available through the links described in the paper. Any additional data or R codes can be requested from the corresponding authors (L.S.).
