# Supplementary material for: Improved spatial ecological sampling using open data and standardization: an example from malaria mosquito surveillance
Source: J R Soc Interface. 2019 Apr 10;16(153):20180941. doi: 10.1098/rsif.2018.0941 (PMC6505554; doi:10.1098/rsif.2018.0941)
Supplement: Electronic Supplementary Material [file rsif20180941supp1.pdf]

Improved spatial ecological sampling using open data and standardization: an example from malaria mosquito surveillance

Luigi Sedda, Eric R. Lucas, Luc S. Djogbénou, Ako V.C. Edi, Alexander Egyir-Yawson, Bilali I. Kabula, Janet Midega, Eric Ochomo, David Weetman and Martin J. Donnelly

Journal of the Royal Society Interface

APPENDICES

Contents

Appendix A: Study sites.. 2

Appendix B: Clustering algorithm. 5

Appendix C: Experimental and fitted variogram for the AIRS Migori data.. 6

Appendix D: Wilk's lambda plot for the ecological classification in each of the sites. 7

Appendix E: Ecological classification table.. 10

Appendix F: Classification and uncertainty map for each site. 11

Appendix G: Effect of stratification on sample size and on the improvement of mosquito abundance model. 14

Appendix A: Study sites.

Aboude

Aboude, Southern Côte d'Ivoire, is located in the evergreen forest zone with altitude between 30 and 100 m above sea level. The climate is characterised by four seasons: a long rainy season (April-July), a short dry season (August-September), a short rainy season (October-November) and a long dry season (December to March). Average temperature is around 27°C and average rainfall of 120mm. Relative humidity ranges from 70 to 85%. The hydrographic network of the region is very diversified and characterized by the presence of the Bandama and the N'zi Rivers with several streams. The primary activity of the rural population is agriculture with mainly cocoa, rubber, vegetable and irrigated rice fields with large use of pesticides. Malaria transmission occurs during the rainy seasons, between April and November [1] but insecticide resistance has not been documented to date.

Grand Popo

The study site in Benin is in the southwestern coastal part of the country. Elevation ranges from 0m to 70m above sea level. The average temperature is 28.9°C, average relative humidity is 76% with average annual rainfall of 190mm. The rainy season is characterized by abundant rains during April to July, and a lower amount of rain from September to October. The area is mostly urban and cultivated, and use of pesticide is common. Studies have been published on malaria incidence and bednet use [2-4], however there are no studies on mosquito species distribution or insecticide resistance.

Malindi

The study site contains the large town of Malindi with approximately 210,000 inhabitants. The climate is tropical, a cooler season from June to September, with daytime temperatures around 27-28 °C, is followed by a hotter and humid season from November to April, with daytime temperatures above 30 °C. Relative humidity ranges between 80-85%. Malindi is comprised of commercial and residential areas, agricultural and undeveloped areas, and hotels and stores along the coast. Tourism, retail, fishing, and trading are the major economic activities. This area is within Kenya's endemic malaria zone with all-year risk of malaria transmission [5]. The major malaria control intervention in Malindi is the use of pyrethroid treated bednets. Studies to detect insecticide resistance show suspected *Anopheles* resistance to pyrethroids [6, 7].

Migori

Migori is located in western Kenya, about 50km from Lake Victoria and with elevation ranging from 1,200m to 1,500m above sea level. The average annual temperature is 21°C, and average relative humidity is 65% with average annual rainfall of 1,000 -1,800mm. The area experiences long rains from April to June and short rains from September to October. The land is mainly used for cultivation and grazing. There are some studies on malaria burden from the area [8, 9], but none on mosquito abundance or insecticide resistance even if indoor residual spraying is taking place.

Muleba

Muleba is in the Kagera region of northwest Tanzania on the western shore of Lake Victoria. The district lies at 1,100-1,600m above sea level. There are two rainy seasons: "long rains" in March – June (average monthly rainfall 300 mm) and "short rains" in October-December (average monthly rainfall 160 mm). Average annual temperature is 21°C (with minimum- maximum range of 15°C-28°C) and average relative humidity of 66%. The area is mainly rural and is used for agriculture. Malaria transmission occurs throughout the year and peaks after the rainy seasons. The

predominant malaria vectors are *Anopheles gambiae s.s.* and *An. arabiensis*, in which pyrethroid resistance has been detected [10].

Obuasi

Obuasi is located in the southern part of the Ashanti region of Ghana about 64 km south-west of the regional capital Kumasi. The area has an undulating terrain with most of the hills rising above 500 meters above sea level and vegetation characteristic of the moist semi-deciduous forest type. The climate is semi-equatorial and characterised by two rainy seasons. The first season starts from March and ends in July and the second from September to November. The mean annual rainfall ranges between 125mm and 175 mm, while the mean average annual temperature is 25.5 °C and relative humidity 75% – 80% in the wet season. Agricultural activities in the area include crop farming, livestock rearing, tree planting and fish farming. Mining and quarry forms the second largest industrial activity in the municipality and creates potential mosquito breeding sites all year round. Resistance to multiple insecticides in Obuasi has been documented in *Anopheles gambiae and An. funestus* mosquitoes [11-13].

References

1 Assi, S. B., Aba, Y. T., Yavo, J. C., Nguessan, A. F., Tchiekoi, N. B., San, K. M., Bissagnene, E., Duparc, S., Lameyre, V., Tanoh, M. A. 2017 Safety of a fixed-dose combination of artesunate and amodiaquine for the treatment of uncomplicated Plasmodium falciparum malaria in real-life conditions of use in Cote d'Ivoire. *Malaria J*. **16**, (10.1186/s12936-016-1655-1)

2 Damien, G. B., Djenontin, A., Rogier, C., Corbel, V., Bangana, S. B., Chandre, F., Akogbeto, M., Kinde-Gazard, D., Massougbodji, A., Henry, M. C. 2010 Malaria infection and disease in an area with pyrethroid-resistant vectors in southern Benin. *Malaria J*. **9**,

3 Moiroux, N., Boussari, O., Djenontin, A., Damien, G., Cottrell, G., Henry, M. C., Guis, H., Corbel, V. 2012 Dry Season Determinants of Malaria Disease and Net Use in Benin, West Africa. *Plos One*. **7**,

4 Djenontin, A., Bio-Bangana, S., Moiroux, N., Henry, M. C., Bousari, O., Chabi, J., Osse, R., Koudenoukpo, S., Corbel, V., Akogbeto, M.*, et al.* 2010 Culicidae diversity, malaria transmission and insecticide resistance alleles in malaria vectors in Ouidah-Kpomasse-Tori district from Benin (West Africa): A pre-intervention study. *Parasite Vector*. **3**,

5 Division of Malaria Control. Towards a Malaria-Free Kenya: National Malaria Strategy 2009–2017. In: M. o. P. H. a. Sanitation, ed. Kenya: Ministry of Public Health and Sanitation 2009:116.

6 Kawada, H., Futami, K., Komagata, O., Kasai, S., Tomita, T., Sonye, G., Mwatele, C., Njenga, S. M., Mwandawiro, C., Minakawa, N.*, et al.* 2011 Distribution of a Knockdown Resistance Mutation (L1014S) in Anopheles gambiae s.s. and Anopheles arabiensis in Western and Southern Kenya. *Plos One*. **6**,

7 Ondeto, B. M., Nyundo, C., Kamau, L., Muriu, S. M., Mwangangi, J. M., Njagi, K., Mathenge, E. M., Ochanda, H., Mbogo, C. M. 2017 Current status of insecticide resistance among malaria vectors in Kenya. *Parasite Vector*. **10**,

8 Okoyo, C., Mwandawiro, C., Kihara, J., Simiyu, E., Gitonga, C. W., Noor, A. M., Njenga, S. M., Snow, R. W. 2015 Comparing insecticide-treated bed net use to Plasmodium falciparum infection among schoolchildren living near Lake Victoria, Kenya. *Malaria J*. **14**,

9 Marube, E., Chahale, T., Onyando, B., Onditi, S., Clark, T., Evance, I., Mwinga, R. D., Martin, T., Kolek, C. 2017 Improvements in Quality of Malaria Case Management through County Referral Hospital Medicines and Therapeutics Committees in Kenya: The Migori County Experience. *Am J Trop Med Hyg*. **97**, 335-336.

10 Protopopoff, N., Matowo, J., Malima, R., Kavishe, R., Kaaya, R., Wright, A., West, P. A., Kleinschmidt, I., Kisinza, W., Mosha, F. W.*, et al.* 2013 High level of resistance in the mosquito

Anopheles gambiae to pyrethroid insecticides and reduced susceptibility to bendiocarb in north-western Tanzania. *Malaria J*. **12**,
11 Riveron, J. M., Osae, M., Egyir-Yawson, A., Irving, H., Ibrahim, S. S., Wondji, C. S. 2016 Multiple insecticide resistance in the major malaria vector Anopheles funestus in southern Ghana: implications for malaria control. *Parasit Vectors*. **9**, 504. (10.1186/s13071-016-1787-8)
12 Hunt, R. H., Fuseini, G., Knowles, S., Stiles-Ocran, J., Verster, R., Kaiser, M. L., Choi, K. S., Koekemoer, L. L., Coetzee, M. 2011 Insecticide resistance in malaria vector mosquitoes at four localities in Ghana, West Africa. *Parasite Vector*. **4**, 107.
13 Okoye, P. N., Brooke, B. D., Koekemoer, L. L., Hunt, R. H., Coetzee, M. 2008 Characterisation of DDT, pyrethroid and carbamate resistance in Anopheles funestus from Obuasi, Ghana. *Trans R Soc Trop Med Hyg*. **102**, 591-598. (10.1016/j.trstmh.2008.02.022)

Appendix B: Clustering algorithm.

The Quadratic Discriminant Analysis (QDA) has been embedded into an algorithm that determines the optimal number of ecological classes and their geographic delimitation for each area. The algorithm steps are the follows:

Initialization

i. Define the initial number of classes, N_0. Our initial choice has been N_0 = the number of land cover classes in the area. This decision is made on the assumption that mosquito distribution is significantly predicted by land use and land cover. The co-variates are all the environmental variables described in the methods of the manuscript.

Splitting algorithm

ii. QDA is applied to N_0 classes in the first iteration, otherwise to N_j.

iii. The class with lowest probability is then split into two sub-classes of similar size based on the criterion of minimum intra-class variance. At the iteration, *j*, the number of classes is $N_j = N_{j-1}+1$.

iv. Repeat ii and iii until N_j is equal to a maximum number of classes, here fixed to 8.

Merging algorithm

v. Set *j*=1

vi. Starting from N_0, merge the two classes with the largest probability that members belong to both classes. At the iteration, *j*, the number of classes is $N_j = N_{j-1}-1$.

vii. Apply QDA to N_j classes

viii. Repeat vi and vii until N_j is equal to a minimum number of classes, here fixed to 2.

Selection of the optimal number of classes

ix. The optimal number, N*,of classes is selected based on the Wilk's criterion [1]. The largest reduction in the Wilk's criterion between two consecutive classes (equivalent to a sharp decline below the trend in the graph plotting Wilk's Lambda on the y-axes and number of classes in the x-classes) indicates the optimal number of classes.

Classification

x. In the final step, all the points are classified in one of the N* classes. Uncertainty is measured as the sum of the probabilities that a point belongs to any of the other classes.

The Wilks' criterion is based on the following general equation:

$$\mathbf{T} = \mathbf{W} + \mathbf{B} \qquad \text{(B1)}$$

where **T** is the total sums of squares and products matrix, **W** is the total sums of squares and products within groups and **B** is the total sums of squares and products between groups. The Wilks' criterion or Wilks' Lambda (*L*) is the ratio of the determinants of **W** and **T**:

$$L = \frac{|\mathbf{W}|}{|\mathbf{T}|} \qquad \text{(B2)}$$

therefore, minimizing *L* is equivalent to minimising |**W**|.

References

1 el Ouardighi, A., el Akadi, A., Aboutajdine, D. Year Feature Selection on Supervised Classification Using Wilks Lambda Statistic. 2007 International Symposium on Computational Intelligence and Intelligent Informatics; 2007; Agadir, Morocco: IEEE; 2007. p. 51-55.

Appendix C: Experimental and fitted variogram for the AIRS Migori data.

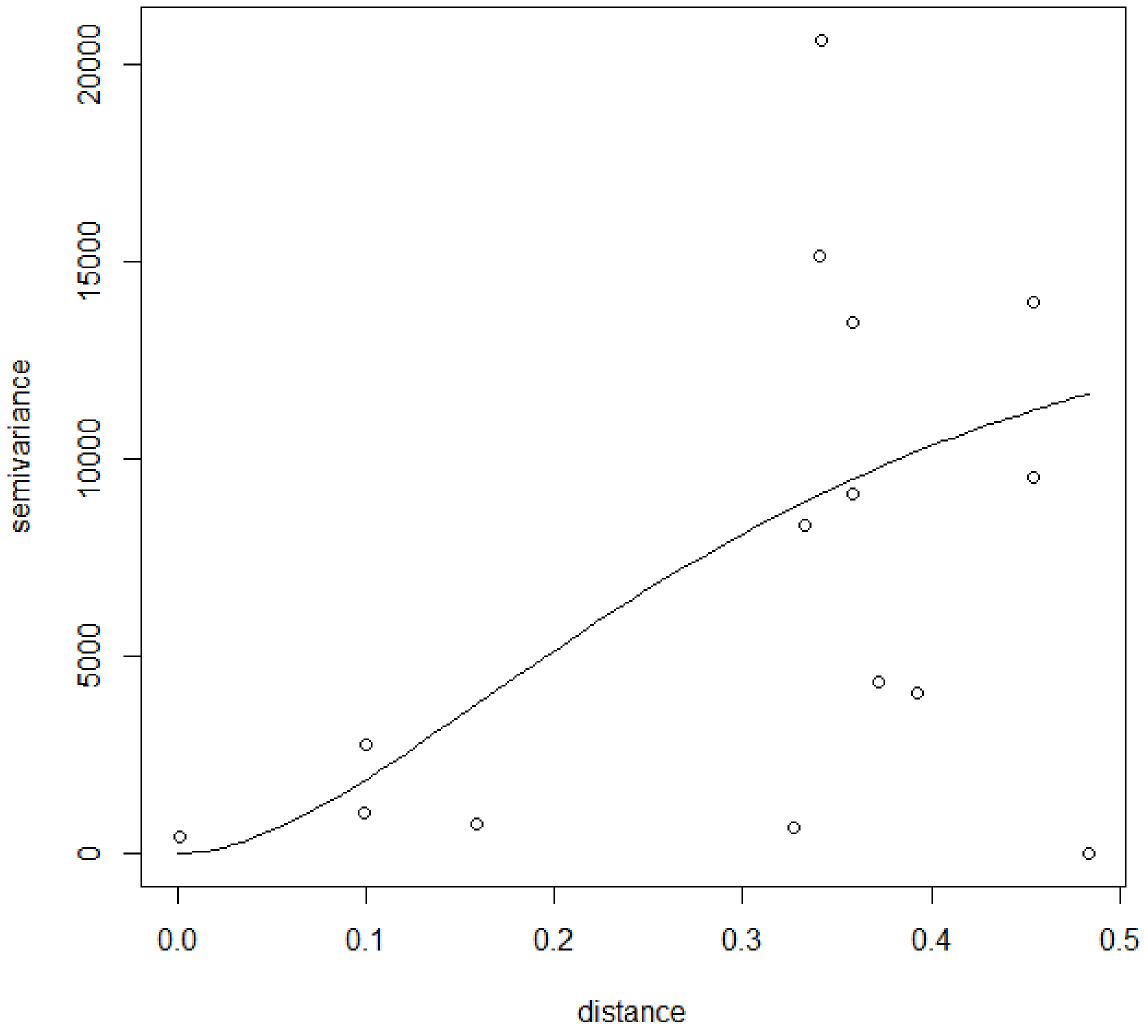

Figure C1. Variogram cloud (points) and fitted correlation function (Matern, with parameters described in the text) for the AIRS mosquito sampling locations in Migori.

Appendix D: Wilk's lambda plot for the ecological classification in each of the sites.

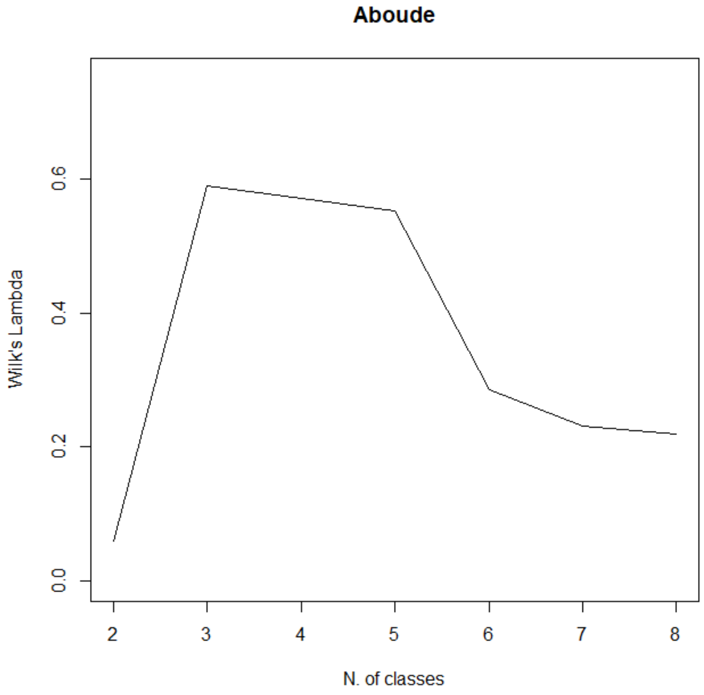

Figure D1. Wilk's Lambda criterion for Aboude (Ivory Coast).

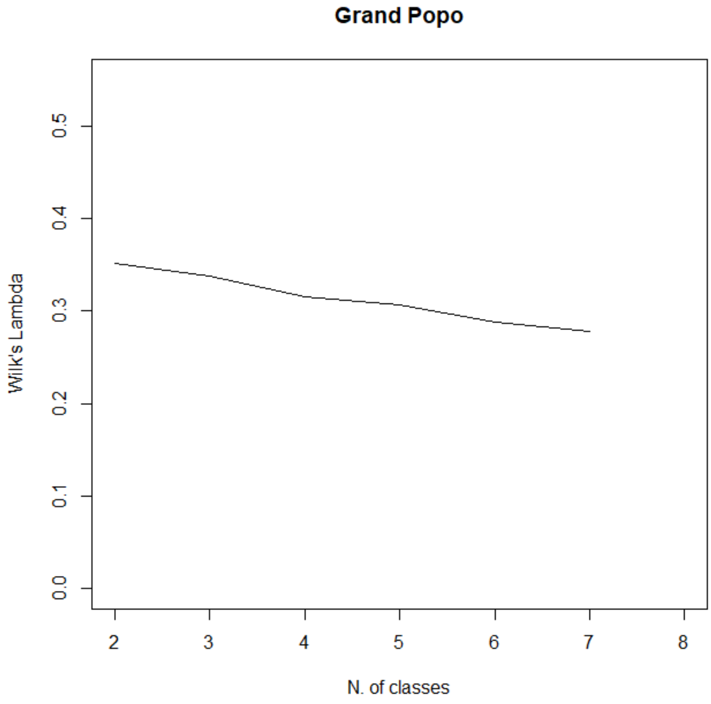

Figure D2. Wilk's Lambda criterion for Grand Popo (Benin).

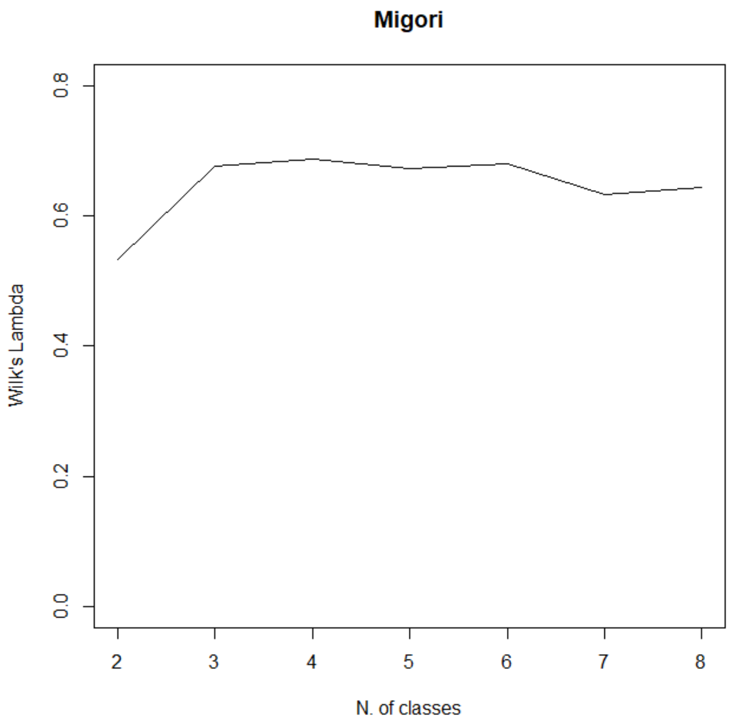

Figure D3. Wilk’s Lambda criterion for Migori (West Kenya).

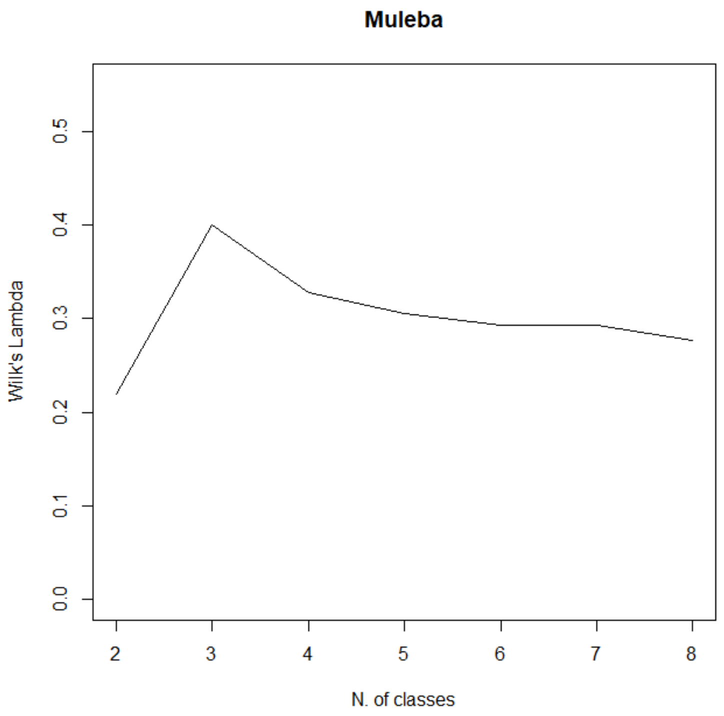

Figure D4. Wilk’s Lambda criterion for Muleba (Tanzania).

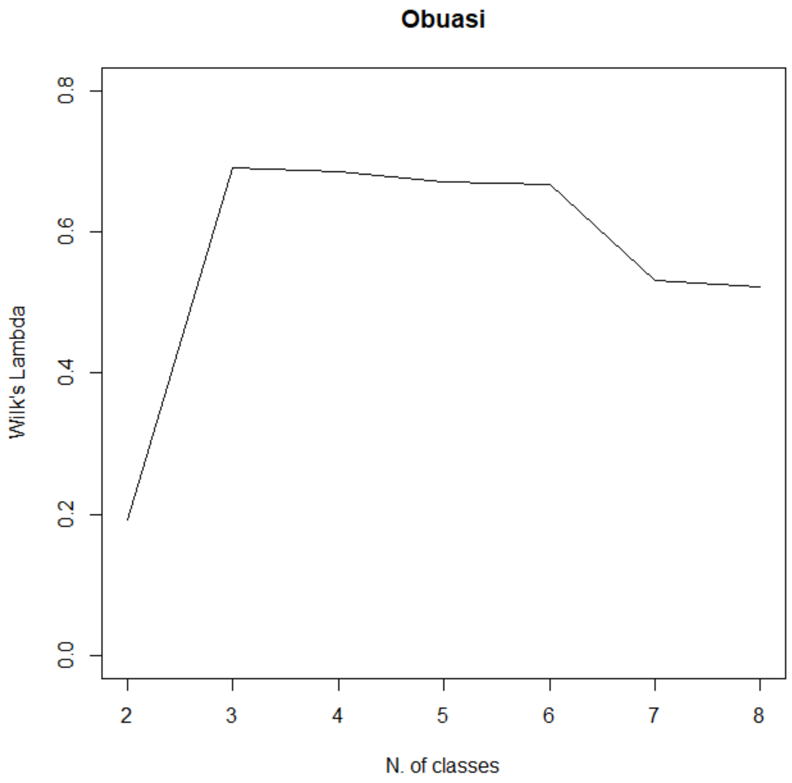

Figure D5. Wilk’s Lambda criterion for Obuasi (Ghana).

Appendix E: Ecological classification table.

Table E1. Classes delineated by ecological classification (described in Appendix B): description, location and colour used in the maps.

Class	Description	Colour
**10**	**Cultivated land**. **Medium** ET mean and variance, EVI mean, variance and amplitude, Temp mean, variance and amplitude, Precipitation; **High** ET amplitude and Elevation.	Mango
**15**	Mixture of **Cultivated land** and **Forest**. **Low** ET mean, EVI variance and Temp mean. **Medium** EVI mean, elevation and Precipitation.	Red
**20**	**Forest**. **Medium** ET mean amplitude and variance, EVI mean, variance and amplitude, Temp mean and variance, Elevation and Precipitation; **High** Temp amplitude.	Dark Green
**25**	Mixture of **Cultivated land, Shrubland** and **Wetland**. **Low** ET mean, EVI variance and Temp mean. **Medium** Elevation and Precipitation.	Orange
**35**	Mixture of **Shrubland** and **Grassland**. **Low** Temp amplitude and Precipitation; **Medium** ET mean and variance, EVI mean, variance and amplitude, Temp mean and variance; **High** ET amplitude.	Light green
**45**	Mixture of **Shrubland, Grassland** and **Wetland**. **Low** EVI amplitude, Temp variance and Precipitation; **Medium** ET mean and amplitude, EVI mean and variance, Temp mean and variance; **High** Temp mean and ET variance.	Pink
**55**	Mixture of **Urban, Forest, Wetland** and **Grassland**. **Low** Temp mean; **Medium** ET mean, EVI mean and variance, and Precipitation; **High** Elevation.	Yellow
**60**	**Wetland**. **Low** ET mean, EVI mean and variance; **Medium** Temp mean and Elevation; **High** Precipitation.	Navy
**65**	Mixture of **Urban, Tundra, Wetland, Water bodies** and **Grassland**. **Low** ET variance and amplitude; **Medium** Elevation; **High** ET mean, EVI mean, variance and amplitude, Temp amplitude and Precipitation.	Purple
**75**	Mixture of **Water bodies** and **Urban**. **Low** ET mean, EVI mean and amplitude, Temp variance and Precipitation; **Medium** ET amplitude and EVI variance; **High** ET variance and Temp mean.	Sky
**85**	Mixture of **Grassland, Water bodies** and **Urban**. **Low** ET variance and amplitude, and Elevation; **Medium** Temp mean; **High** ET mean, EVI mean, variance and amplitude, Temp variance and Precipitation.	Brown
**95**	Mixture of **Wetland, Water bodies** and **Urban**. **Low** EVI mean and amplitude, and Temp amplitude; **Medium** ET mean, EVI variance, Temp variance and Precipitation; **High** ET variance and amplitude, and Temp mean.	Grey

*Low, values lower than 25% quartile; Medium, values between 25% and 75% quartiles; High, values larger than 75% quartile.

Appendix F: Classification and uncertainty map for each site.

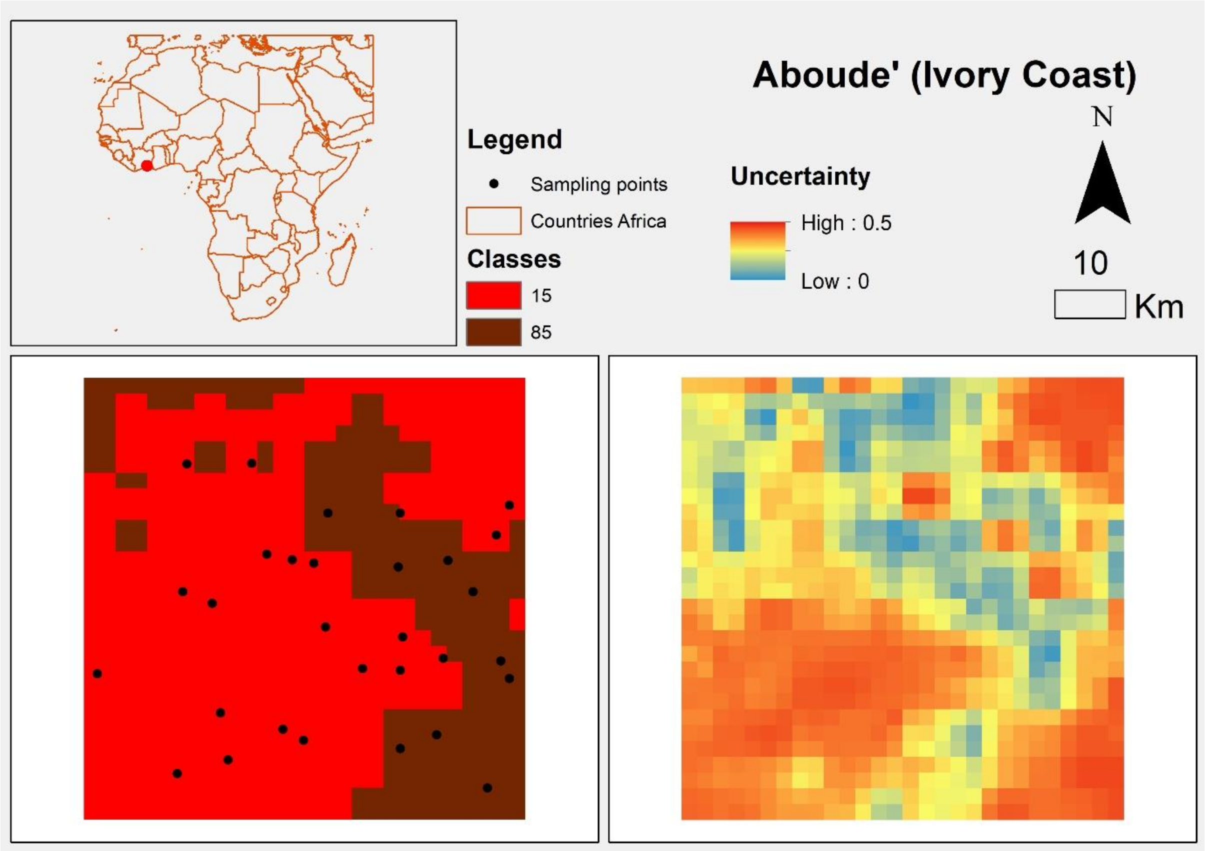

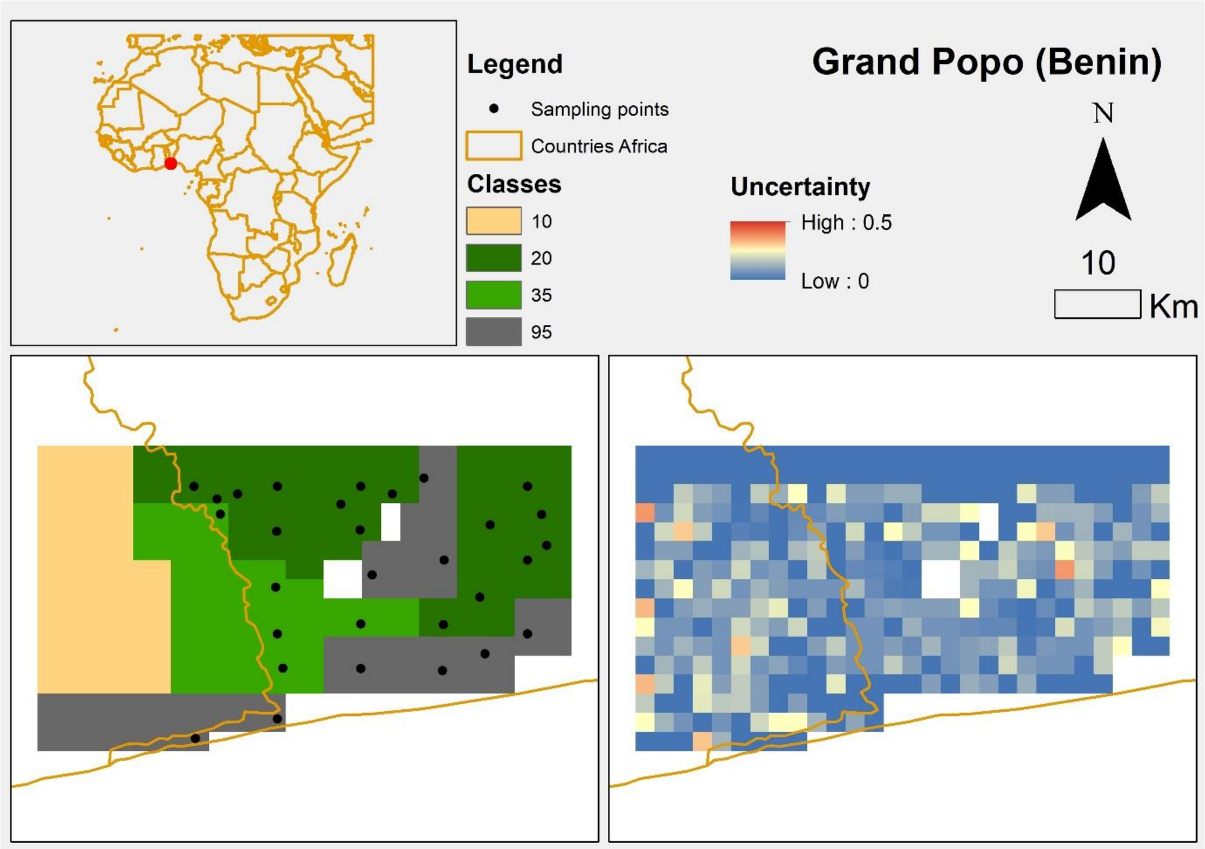

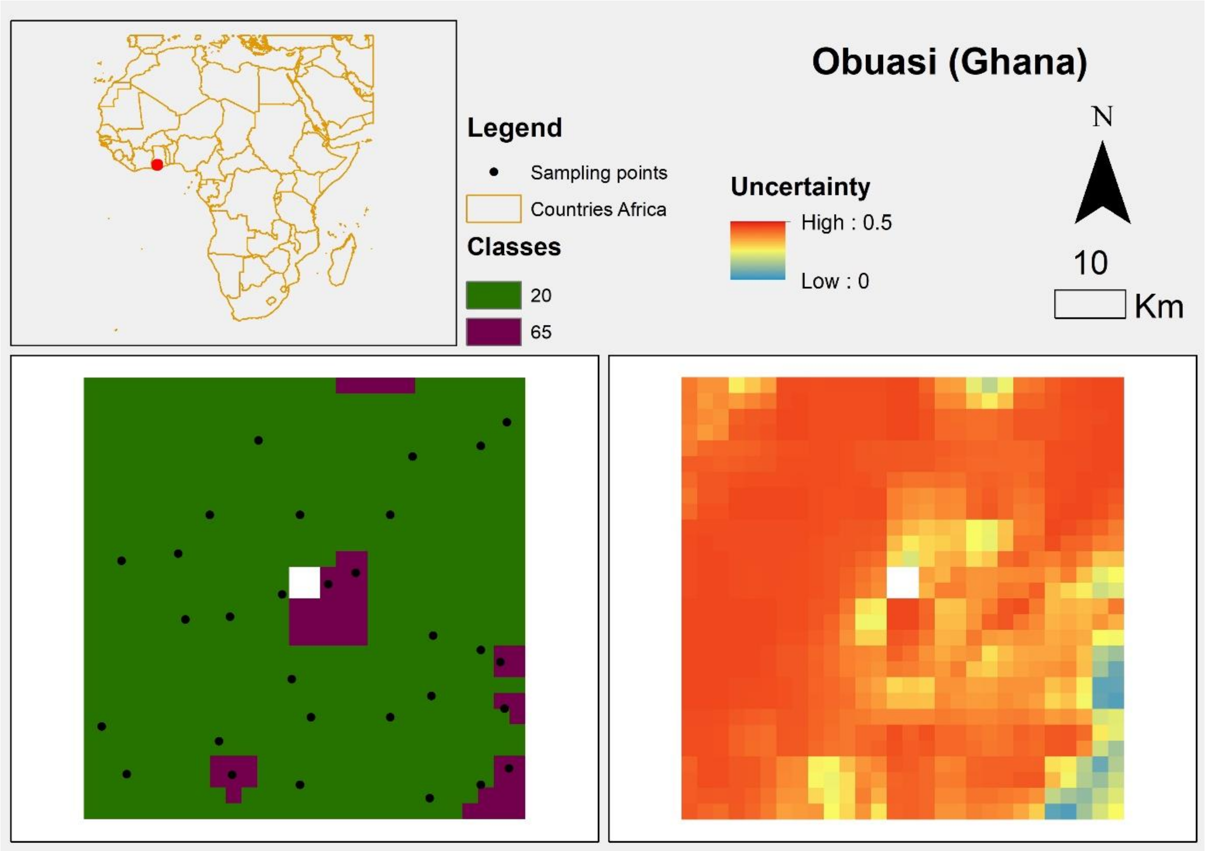
Obuasi (Ghana)
Legend
Sampling points
Countries Africa
Classes
20
65
Uncertainty
High : 0.5
Low : 0
N
10
Km

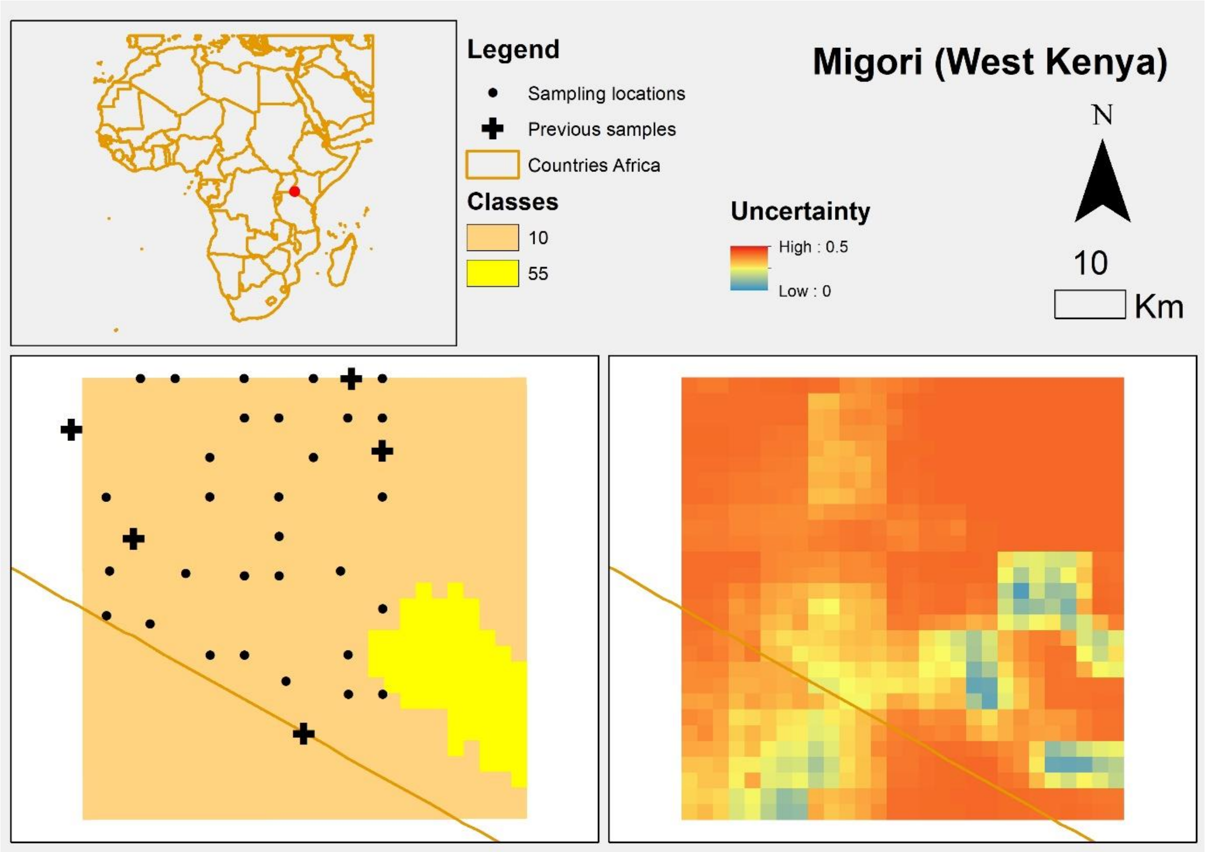
Legend
Sampling locations
Previous samples
Countries Africa
Classes
10
55
Migori (West Kenya)
Uncertainty
High : 0.5
Low : 0
N
10
Km

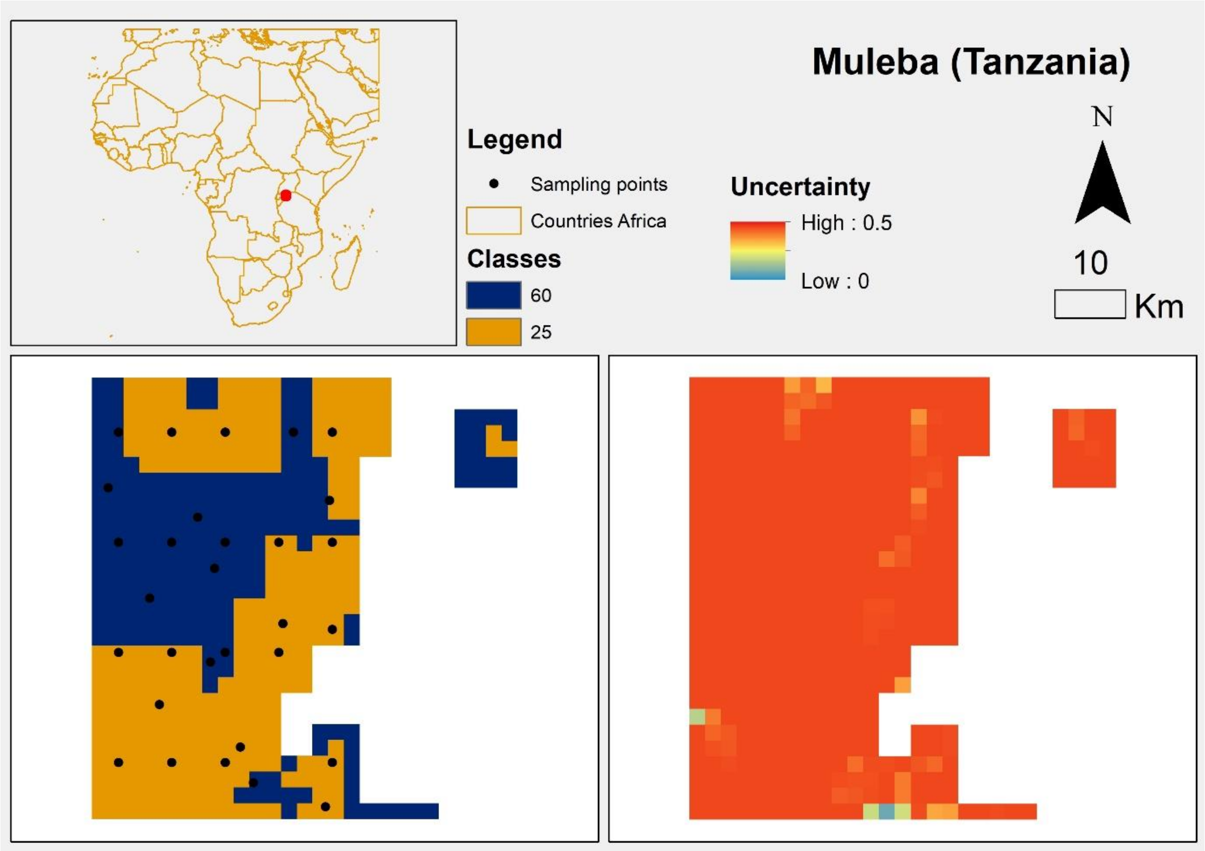

Muleba (Tanzania)
Legend
Sampling points
Countries Africa
Classes
60
25
Uncertainty
High : 0.5
Low : 0
N
10
Km

Appendix G: Effect of stratification on sample size and on the improvement of mosquito abundance model.

Table G1. 95% Confidence interval (CI) of the rate ratio between λ_1 and λ_2 (Poisson distribution rate parameters from mosquito counts in the full survey and mosquito counts in a sub-sample of locations respectively), where λ_2 is calculated for each sample size. The sample size refers to each strata for a total of 2*4, 3*4, 4*4 and 5*4 locations, where 4 is the number of strata. In the case of complete random sampling (last 3 rows), then the 2*4, 3*4, 4*4 and 5*4 are the number of locations sampled independently from the strata.

	CI	*2*	*3*	*4*	*5*
Stratified	*0.025*	0.94	0.91	0.86	0.85
	0.5	1.05	0.99	0.93	0.9
	0.975	1.16	1.08	1	1.07
Random	*0.025*	1.11	1.11	1.03	1.04
	0.5	1.25	1.21	1.11	1.07
	0.975	1.4	1.33	1.21	1.12

Table G2. ANOVA analyses of Poisson generalised linear models for female (F), male (M) and total (F+M) mosquitoes of *An gambiae* and *An. funestus*. Residual deviance is in % of the Null deviance.

Species	*Sex*	*Residual deviance*	*P value*
An. gambie	F	87	2.2 e-16
	M	89	2.2 e-16
	F+M	88	2.2 e-16
An. funestus	F	88	2.2 e-16
	M	93	2.2 e-16
	F+M	89	2.2 e-16
